# Distinct Effects of Immunosuppressive Drugs on the Anti-*Aspergillus* Activity of Human Natural Killer Cells

**DOI:** 10.3390/pathogens8040246

**Published:** 2019-11-19

**Authors:** Stanislaw Schmidt, Ralf Schubert, Asuman Demir, Thomas Lehrnbecher

**Affiliations:** 1 Division of Pediatric Hematology and Oncology, Hospital for Children and Adolescents, University Hospital, Goethe University Frankfurt am Main, Theodor-Stern-Kai 7, 60590 Frankfurt, Germany; stanislaw.schmidt@kgu.de (S.S.); asuman.demir@kgu.de (A.D.); 2 Pediatric Pulmonology, Allergology and Cystic Fibrosis, Hospital for Children and Adolescents, University Hospital Frankfurt, Theodor-Stern-Kai 7, 60590 Frankfurt, Germany; ralf.schubert@kgu.de

**Keywords:** *Aspergillus fumigatus*, human Natural Killer cell, immunosuppressive drug, cyclosporin A, methylprednisolone, mycophenolic acid

## Abstract

As the prognosis of invasive aspergillosis remains unacceptably poor in patients undergoing hematopoietic stem cell transplantation (HSCT), there is a growing interest in the adoptive transfer of antifungal effector cells, such as Natural Killer (NK) cells. Because immunosuppressive agents are required in most HSCT recipients, knowledge of the impact of these compounds on the antifungal activity of NK cells is a prerequisite for clinical trials. We, therefore, assessed the effect of methylprednisolone (mPRED), cyclosporin A (CsA) and mycophenolic acid (MPA) at different concentrations on proliferation, apoptosis/necrosis, and the direct and indirect anti-*Aspergillus* activity of human NK cells. Methylprednisolone decreased proliferation and increased apoptosis of NK cells in a significant manner. After seven days, a reduction of viable NK cells was seen for all three immunosuppressants, which was significant for MPA only. Cyclosporin A significantly inhibited the direct hyphal damage by NK cells in a dose-dependent manner. None of the immunosuppressive compounds had a major impact on the measured levels of interferon-γ, granulocyte-macrophage colony-stimulating factor and RANTES (regulated on activation, normal T cell expressed and secreted; CCL5). Our data demonstrate that commonly used immunosuppressive compounds have distinct effects on proliferation, viability and antifungal activity of human NK cells, which should be considered in designing studies on the use of NK cells for adoptive antifungal immunotherapy.

## 1. Introduction

Patients undergoing allogeneic hematopoietic stem cell transplantation (HSCT) are at an increased risk for invasive fungal disease (IFD), with *Aspergillus fumigatus* as the fungal pathogen most commonly isolated [1]. Despite new and potent antifungal agents, morbidity and mortality of invasive aspergillosis in HSCT recipients is unacceptably high, which explains the growing interest in immunotherapeutic approaches, such as adoptively transferring antifungal effector cells or administering of cytokines or interferons in this setting [2,3]. The antifungal host immune response is a complex network consisting of effector cells, such as phagocytes, T cells, and NK cells and soluble mediators which are released by numerous cell populations [4,5]. Human Natural Killer (NK) cells have the potential to kill targets without prior activation, and it has been shown in vitro that NK cells damage fungi of different genera and species [6,7,8,9,10]. NK cells are able to exert direct antifungal activity via cytotoxic molecules, such as perforin, but also modulate the antifungal host response via the release of molecules, such as interferon (IFN)-γ, granulocyte-macrophage colony-stimulating factor (GM-CSF) or RANTES (regulated upon activation, normal T-cell expressed, and secreted; chemokine ligand 5) [11,12,13]. In contrast, the impact of activating and inhibitory NK receptors, such as natural cytotoxicity receptors (NCR) 1-3, CD56, CD16 or killer-immunoglobulin-like receptors (KIRs) on the antifungal activity of NK cells has not been fully characterized to date and has to be addressed in future experiments. This is also the fact for the antifungal activity of the different NK subpopulations, such as cytotoxic CD56^dim^CD16^bright^ and immune regulatory CD56^bright^CD16^dim^ cells [5,14]. The in vitro data are supported by animal models, which clearly demonstrate the importance of NK cell-derived IFN-γ in neutropenic mice with pulmonary aspergillosis, and that the adoptive administration of NK cells results in a benefit [15]. As HSCT recipients often receive immunosuppressive compounds to prevent or to treat graft-versus-host disease (GvHD), and as the anti-tumor properties of NK cells may differ from those against fungi, we investigated the effects of different concentrations of methylprednisolone (mPRED), cyclosporin A (CsA) and mycophenolic acid [MPA as the active metabolite of the pro-drug mycophenolate mofetil (MMF)] on proliferation, viability and on the direct and indirect anti-*Aspergillus* activity of human NK cells. 

## 2. Results

### 2.1. Anti-Aspergillus Activity of Human NK Cells Co-Incubated with Immunosuppressive Agents

**Figure 1:**
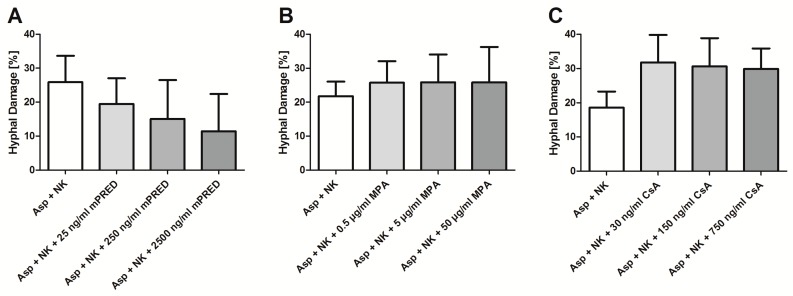
Measured anti-*Aspergillus* activity of human NK cells co-incubated with immunosuppressive compounds. Shown is the net-effect (equivalent to the added effect of NK cells and the immunosuppressive compound) of *Aspergillus fumigatus* hyphae when NK cells were incubated with mPRED (**A**), MPA (**B**) or CsA (**C**), respectively, at various concentrations. The white bars on the left side represent the hyphal damage by NK cells alone. Data were assessed by the XTT assay after 6 hours of incubation. Shown are mean and SEM from at least three independent experiments. NK cells—Natural Killer cells; Asp—*Aspergillus fumigatus*; mPRED—methylprednisolone; MPA—mycophenolic acid; CsA—cyclosporin A.

Immunosuppressive agents per se may exhibit antifungal activity [16]. Therefore, when co-incubating *A. fumigatus* hyphae with both human NK cells and immunosuppressive drugs, the measured hyphal damage represents the net-effect of the hyphal damage mediated by NK cells (treated with an immunosuppressive drug) and the hyphal damage exhibited by the immunosuppressive drug alone. Analyzing this net-effect, a slight decrease in the mean hyphal damage was seen for NK cells treated with mPRED, although this decrease did not reach statistical difference (mean ± SEM: NK cells alone 25.9% ± 7.8%, NK cells + mPRED at 25, 250, and 2500 ng/mL 19.4% ± 7.6%, 15.1% ± 11.4%, and 11.4% ± 11.0%, respectively; Figure 1A). The mean measured hyphal damage of *A. fumigatus* hyphae by NK cells of 18.6% ± 4.7% slightly increased in the presence of CsA at 30, 150, and 750 ng/mL to 31.7% ± 8.1%, 30.6% ± 8.3%, and 29.9% ± 6.0%, respectively (Figure 1C), which did not reach statistical significance.

**Figure 2:**
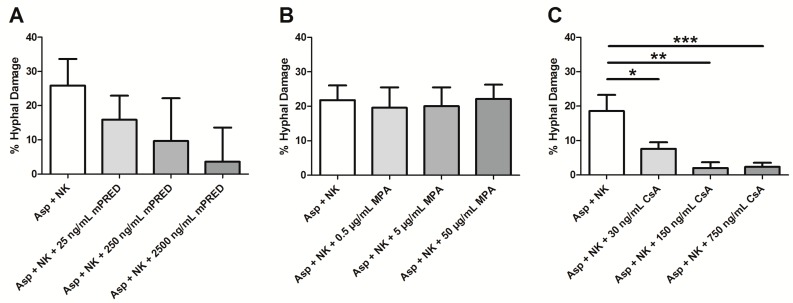
Calculated hyphal damage defined as the combined antifungal effect of NK cells and immunosuppressive agent minus the antifungal effect of the immunosuppressive agent alone. Shown is the effect on *A. fumigatus* hyphae of untreated NK cells alone, and of the calculated hyphal damage of NK cells co-incubated with various concentrations of mPRED (**A**), MPA (**B**) or CsA (**C**), respectively. Shown are mean and SEM from at least three independent experiments. NK cells—Natural Killer cells; Asp—*Aspergillus fumigatus*; mPRED—methylprednisolone; MPA—mycophenolic acid; CsA—cyclosporin A. *: *p* < 0.05; **: *p* < 0.01; ***: *p* < 0.005.

In order to analyze the impact of an immunosuppressive agent on the antifungal activity of NK cells, the difference of the measured hyphal damage mediated by NK cells and the immunosuppressive drug (Figure 1A–C) and the hyphal damage exhibited by the immunosuppressive drug alone (Supplementary Figure S1) has to be calculated. In this analysis, CsA significantly decreased the effect of human NK cells against A. fumigatus hyphae (mean ± SEM: NK cells alone 18.6% ± 4.7% and 2.4% ± 1.2% at the highest concentration of 750 ng/mL of CsA; *p* = 0.041; Figure 2C). As MPA and mPRED alone did not exhibit major damage of A. fumigatus hyphae (Supplementary Figure S1), no significant difference between the measured and calculated hyphal damage by NK cells in the presence or absence of mPRED and MPA, respectively, was seen (Figure 2A,B).

### 2.2. Effect of Immunosuppressive Agents on Apoptosis/Necrosis, Proliferation and Absolute Cell Numbers of Human NK Cells

**Figure 3:**
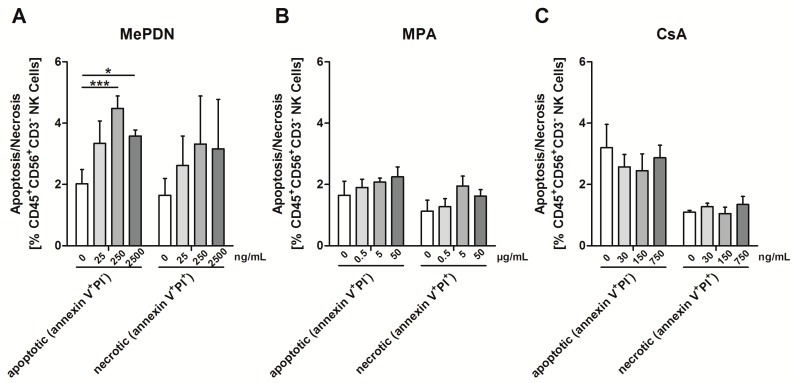
Effect of immunosuppressive compounds on the viability of human NK cells. Percentage of apoptotic (on the left) and necrotic (on the right) NK cells incubated alone or with various concentrations of mPRED (**A**), MPA (**B**) or CsA (**C**) assessed by means of Annexin V and propidium iodide (PI) staining after 24 h. Shown are mean and SEM from at least three independent experiments. NK cells—Natural Killer cells; Asp—*Aspergillus fumigatus*; mPRED—methylprednisolone; MPA—mycophenolic acid; CsA—cyclosporin A; PI—propidium iodite. *: *p* < 0.05; **: *p* < 0.005.

Compared to untreated controls, mPRED at higher concentrations significantly increased apoptosis of IL-2 stimulated human NK cells (mean ± SEM; mPRED at 0, 25, 250, and 2500 ng/mL, 2.0% ± 0.5%, 3.3% ± 0.7% (not significant), 4.5% ± 0.4% (*p* = 0.005) and 3.6% ± 0.2% (*p* = 0.014); Figure 3A). In contrast, MPA and CsA did not significantly alter the apoptosis and necrosis of NK cells (Figure 3B,C).

**Figure 4:**
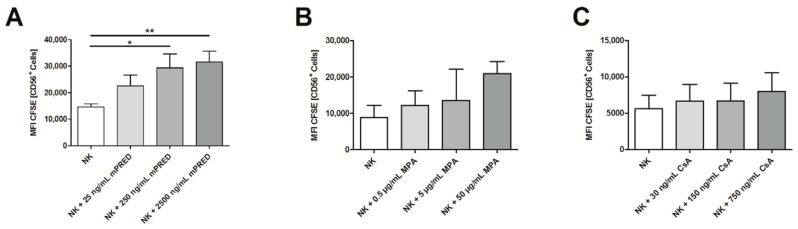
Effect of immunosuppressive compounds on the proliferation of human NK cells. Mean fluorescence intensity (MFI) of CFSE as a measure of proliferation with higher MFI values indicating decreased proliferation was assessed by means of the carboxy-fluorescein-diacetate-succinimidyl-ester labelling assay of NK cells incubated with various concentrations of mPRED (**A**), MPA (**B**) or CsA (**C**) determined on for seven days. Shown are mean and SEM from at least three independent experiments. *: *p* < 0.05; **: *p* < 0.01.

In addition, mPRED at higher concentrations significantly decreased cell proliferation of NK cells, indicated by significantly higher mean fluorescence intensity (MFI) of CFSE in the NK cell population after seven days (mean ± SEM: mPRED at 0, 25, 250, and 2500 ng/mL 14,627 ± 1171, 22,546 ± 4167 (not significant), 29,400 ± 5194 (*p* = 0.032), and 31,546 ± 4032 (*p* = 0.007), respectively; Figure 4A and Supplementary Figure S1). In contrast, MPA and CsA did not significantly inhibit proliferation (Figure 4B,C). 

**Figure 5:**
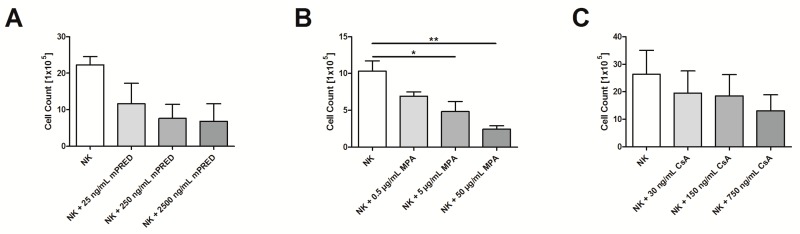
Immunosuppressants reduce the cell number of viable NK cells. Cell count of viable NK cells incubated with various concentrations of mPRED (**A**), MPA (**B**) or CsA (**C**) for seven days as assessed by microscopy. Shown are mean and SEM from at least three independent experiments. *: *p* < 0.05; **: *p* < 0.01.

Only MPA at higher dosages significantly decreased the cell count of viable NK cells determined after seven days (mean ± SEM: control 10.3 × 10^5^ ± 1.4 × 10^5^, MPA at 0.5 µg/mL, 5 µg/mL and 50 µg/mL 6.9 × 10^5^ ± 0.6 × 10^5^ (not significant), 4.8 × 10^5^ ± 1.4 × 10^5^ (*p* = 0.0482), and 2.4 × 10^5^ ± 0.5 × 10^5^ (*p* = 0.0058), respectively (Figure 5B), whereas, the impact of mPRED and CsA on the cell count was not significant (Figure 5A,C).

### 2.3. Effect of the Immunosuppressive Drugs on the Cytokine Profile of Human NK Cells 

**Figure 6:**
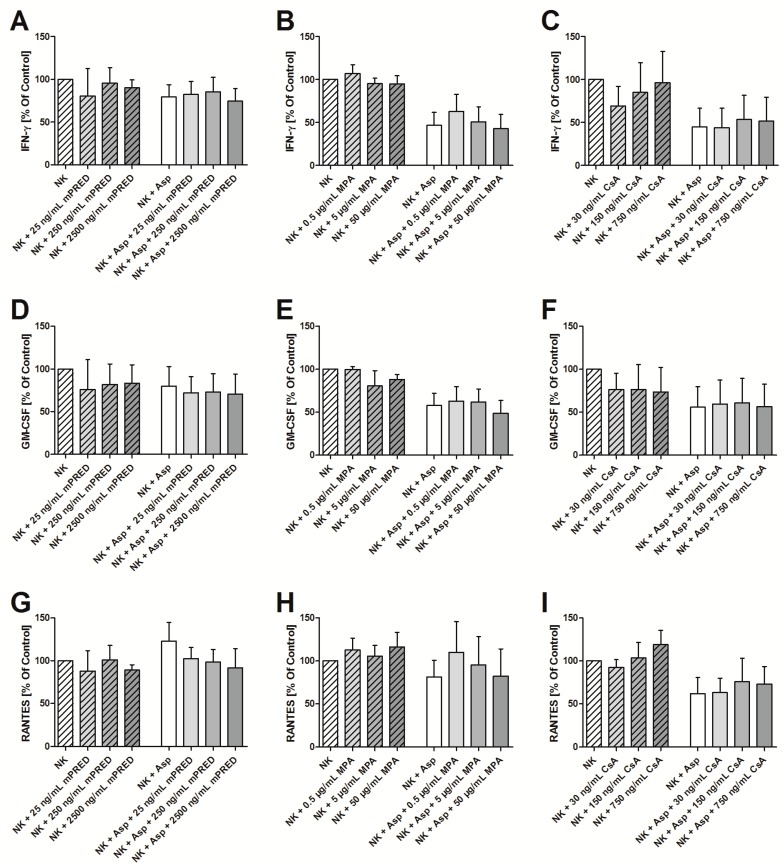
Immunosuppressive drugs have no impact on the cytokine levels in the supernatant of NK cells incubated in the presence or absence of *A. fumigatus* hyphae. Levels of IFN-γ (**A–C**), GM-CSF (**D–F**) and RANTES (regulated on activation, normal T cell expressed and secreted; CCL5) (**G–I**) in the supernatant of human NK cells were assessed by means of the Cytometric Bead Array after for six hours of incubation. The cytokine level of NK cells incubated alone (without the fungus and without any immunosuppressant) was set as 100%. Shown are mean and SEM from at least three independent experiments. NK cells–Natural Killer cells; Asp—*A. fumigatus*; mPRED—methylprednisolone; MPA—mycophenolic acid; CsA—cyclosporin A; IFN-γ—interferon-gamma; GM-CSF—granulocyte-macrophage colony-stimulating factor.

NK cells modulate the antifungal host response by secreting interferons and cytokines, which are able to stimulate the production and function of immune cells, such as neutrophils and T cells. None of the immunosuppressive compounds tested, mPRED, MPA and CsA, exhibited a significant impact on the measured mean levels of IFN-γ (Figure 6A–C), GM-CSF (Figure 6D–F) and RANTES (Figure 6G–I) of IL-2 stimulated human NK cells in the presence or absence of A. fumigatus, respectively. In addition, no significant impact of immunosuppressive compounds was seen on the concentration of perforin in the supernatant (data not shown).

## 3. Discussion

Children and adults receiving an allogeneic HSCT have a significant risk for invasive aspergillosis. Unfortunately, despite the development of new antifungal agents, patients suffering from invasive aspergillosis still have significant morbidity and mortality [1,2,3]. Therefore, immunotherapeutic approaches, such as the use of immune effector cells or cytokines and interferons, are of interest, as they may have an impact on outcome in this setting. Although phagocytes, such as granulocytes are considered as the most important cells for the antifungal host defence, it has been shown that human NK cells kill A. fumigatus in vitro, and the adoptive transfer of functionally active NK cells supports the clearance of pulmonary aspergillosis in immunocompromised mice [6,7,15]. The administration of immunosuppressive drugs is standard of care in allogeneic HSCT recipients for the prevention or for treatment of GvHD, which is another potential but important complication in these patients. Immunosuppressive drugs negatively affect various pathways of the immune system, which, however, are important in the antifungal host response. For example, mPRED impairs phagocytosis of Aspergillus conidia by neutrophils [17], modulates cytokine production, induces apoptosis and inhibits early TCR signaling events in T cells [18]. Mycophenolate mofetil (MMF), the pro-drug of mycophenolic acid (MPA), inhibits proliferation of T and B cells by depleting guanosine nucleotides, and thereby suppresses cell-mediated immune responses and antibody formation. Mycophenolic acid also has a negative impact on the glycosylation and expression of adhesion molecules, and impairs the recruitment of immune effector cells into sites of inflammation [19]. Cyclosporin A acts as an inhibitor of calcineurin, an enzyme regulating the translocation of NF-AT transcription factors into the cell nucleus where they are activated. Thereby, CsA inhibits transcription of cytokine genes, such as IL-2 and IL-4, and blocks signaling pathways important for T cell activation [20]. As knowledge of the effect of commonly used immunosuppressive compounds on NK cells is an absolute need for studies on these cells as antifungal immunotherapy in HSCT recipients, we investigated the impact of mPRED, CsA and MPA on viability and antifungal properties of NK cells.

Our results demonstrate that mPRED increases apoptosis and has a strong negative impact on the proliferation of human NK cells, whereas, no significant decrease in the anti-Aspergillus activity of human NK cells was observed. In contrast, it has been reported that mPRED decreases both the natural and receptor-mediated cytotoxicity of NK cells against various tumor cells lines [21,22]. The latter observation was explained by the inhibition of the surface expression of the Natural Cytotoxicity Receptors (NCR) NKp30 and NKp44. Despite there are studies suggesting that NKp30 plays a role in recognition of C. albicans and C. neoformans [23], the importance of NCRs in the anti-Aspergillus activity of human NK cells is unclear to date. Although the cytotoxic protein perforin is thought to play a role in the damage of A. fumigatus, C. neoformans and other fungi [6,24,25,26], we could not detect a significant effect of the mPRED on the concentration of perforin in the supernatant of NK cells. 

Our results did not reveal any major effect of MPA on the anti-Aspergillus activity of human NK cells, corroborating studies investigating the effect of short-term MPA treatment (<24 h) on cytotoxicity of IL-2 activated NK cells towards tumor cells [27]. Similar observations were made by Ohira et al., demonstrating that MMF co-incubation for up to 7 days does not alter the expression of NKp30, NKp44 or NKG2D on IL-2 activated NK cells [28], whereas, long-term incubation with MPA for 9 days seems to reduce cytotoxicity and corresponds with inhibited acquisition of NCRs [27]. The results suggest that the inhibitory effect of MMF/MPA on IL-2 activated NK cell function is a rather late event. In contrast, Pradier et al. reported on a significant reduction of anti-tumor activity by NK cells which were pre-stimulated overnight with IL2 (50 U/mL) and IL12 (0.5 ng/mL) indicating that the pre-stimulation modifies the effect of immunosuppressive agents on NK cells [22].

Cyclosporin A has inhibitory effects on fungal growth and also exhibits hyphal damage [29] (Supplementary Figure S1). This antifungal effect by CsA compensates the agent´s functional impairment of NK cells in vitro, which explains the fact that co-incubation of Aspergillus hyphae, NK cells and CsA did not result in an alteration of hyphal damage (“net-effect”). Therefore, when deducting the anti-Aspergillus activity of CsA, the calculated data demonstrate that CsA significantly inhibits the anti-Aspergillus activity of human NK cells. Importantly, in cyclophosphamide-treated mice with A. fumigatus infection of the lungs, daily administration of CsA could not rescue the animals [29], underlining that the antifungal effect of CsA does not outweigh its immunosuppressive effect in vivo. Data on the effect of CsA on the activity of human NK cells against other fungi are missing, and the findings in the setting of anti-tumor activity of NK cells are conflicting [22,28,30].

As the antifungal effect of NK cells critically depends on a sufficient effector-to-target-ratio [6], data on the effect of immunosuppressive agents on apoptosis, necrosis, and proliferation, all of which determine the number of viable cells, are important for the use of these cells for adoptive antifungal immunotherapy in HSCT patints. In this regard, co-incubation of NK cells with MPA resulted in a significant decrease of cell numbers. Whereas, mPRED significantly increased apoptosis and necrosis, and also significantly reduced NK cell proliferation, the decrease of viable human NK cells after seven days was not statistically significant. Previous reports demonstrated that various concentrations of mPRED, MPA and CsA led to a decrease of proliferation and NK cell numbers [22,27,28,30], whereas, dexamethasone, another glucocorticosteroid, did not affect NK cell viability [31]. However, it is important to note that in the experiments, different protocols for NK cell pre-stimulation were used. A decrease of NK cell number by CsA in vivo was also described in a mouse model, where both the number of NK cells and lymphocytes were reduced, whereas, the myeloid subset was unaffected [29].

None of the immunosuppressive compounds tested had an impact on the levels of IFN-γ, GM-CSF and RANTES, respectively. This finding was independent of the concentration of the compound used and of the fact whether NK cells and the respective immunosuppressive compound were co-incubated with or without A. fumigatus. On the other hand, as previously reported, A. fumigatus alone up-regulates the production of IFN-γ, but reduces its release, which ultimately results in decreased extracellular levels [32]. The exact mechanism of this phenomenon has not been resolved to date. We focused on the assessment of IFN-γ, GM-CSF and RANTES as they play important roles in the immune modulation by NK cells, but we recognize the fact that the selection of other molecules could have revealed different results. However, similar to our data, 24 h incubation of IL-2 pre-stimulated human NK cells with MPA had minimal effects on the secretion of cytokine and chemokines, such as IFN-γ, IL-6, TNF-α, CXCL8, CCL2, CCL4 and CCL5 [27]. In contrast, co-incubation of NK cells with mPRED, MPA and CsA for at least five days significantly reduced the secretion of IFN-γ [28,33]. The differences might be explained by different pre-stimulation protocols as other groups used 100 U/mL IL-2 for pre-stimulation of NK cells, whereas, our experiments were performed with 1000 U/mL of IL-2. In this regard, the optimal pre-stimulation strategy is not clear to date, and therefore, it is not surprising that combinations of one or more cytokines, including IL-2, IL-12, IL-15, IL-18, and IL-21 have also been used at different dosages. In our study, we used a pre-stimulation protocol that has already been applied in the clinical setting by various groups [34,35,36]. As both the stimulation protocol of NK cells and the time of exposure of NK cells with the respective immunosuppressive drug are critical determinants for the immune response, future studies are warranted to optimize these conditions for each setting. 

In conclusion, our data demonstrate that commonly used immunosuppressive compounds have distinct effects on anti-Aspergillus properties of human NK cells. Although we recognize that our data are derived from in vitro studies, our results show that CsA has major negative effects on the effect of antifungal activity of NK cells, whereas, MPA significantly reduced the number of viable NK cells, which should be considered in designing studies on the use of NK cells for adoptive antifungal immunotherapy.

## 4. Materials and Methods

### 4.1. Preparation of Primary Human NK Cells

Peripheral blood from different anonymized, healthy volunteers (German Red Cross Blood Donor Service Baden-Wuerttemberg-Hessen, Frankfurt, Germany) was used to isolate primary human NK cells by negative selection using the EasySep^®^ Human NK Cell Enrichment Kit (StemCell Technologies, Grenoble, France) [26]. Determination of viability and purity of isolated CD56^+^CD3^-^ NK cells was performed by flow cytometry (Canto II, Beckton Dickinson, San Jose, CA, USA; BD FACSDiva Software v6.1.3, BD Biosciences, San Jose, CA, USA) and revealed that both were ≥90%. Isolated NK cells were cultivated in RPMI (Gibco, Paisley, UK) supplemented with 5% human frozen plasma (German Red Cross Blood Donor Service Baden-Wuerttemberg-Hessen, Frankfurt, Germany) and 1000 U/mL recombinant human interleukin (rhIL)-2 (Novartis, Basel, Switzerland) for up to 10 days. The protocol was approved by the local Ethics committee. 

### 4.2. Preparation of Aspergillus fumigatus

*Aspergillus fumigatus* (strain AF4215, MYA 1163; American Type Culture Collection) was grown on Sabouraud glucose agar plates (BD Bioscience, San Jose, CA, USA) for three days at 37 °C. Conidia were collected in Hanks’ balanced saline solution (Gibco) and filtered through a cell strainer. The estimation of the conidial number was performed microscopically in a Neubauer slide (LO–Laboroptik, Friedrichsdorf, Germany). Resting conidia were immediately used or were stored at 4 °C for a maximum of one week. *A. fumigatus* hyphae were prepared by seeding of resting conidia in flat-bottom cell culture plates (Nunc, Langenselbold, Germany) in Yeast Nitrogen Base (Sigma-Aldrich, Taufkirchen, Germany) medium for 17 h at 37 °C.

### 4.3. Immunosuppressive Compounds

The immunosuppressive agent’s cyclosporin A (CsA; Novartis Pharma, Nürnberg, Germany), methylprednisolone (mPRED; Sanofi Aventis, Frankfurt, Germany), mycophenolic acid (MPA; Sigma-Aldrich, Steinheim, Germany) as the active component of mycophenolate mofetil (MMF), were commercially obtained. All agents were prepared according to the manufacturers´ instructions. Three concentrations of each of the agents were prepared: In addition to the concentration reflecting the common target serum level (e.g., 150 µg/mL for CsA), each compound was tested in a significantly lower and higher concentration. In particular, CsA was used at concentrations of 0.03, 0.15 and 0.75 µg/mL, MPA at 0.5, 5.0 and 50 µg/mL, and methylprednisolone at 0.025, 0.25 and 2.5 µg/mL, respectively [37,38,39,40]. Each experiment has been performed at least three times, and all experiments have been performed independently from each other using NK cells from a different donor.

### 4.4. Assessment of the Antifungal Activity of NK Cells

The direct anti-*Aspergillus* activity of human NK cells in the presence or absence of immunosuppressive compounds was analyzed using a colorimetric assay using XTT (2,3-bis[2-methoxy-4-nitro-5-sulphenyl]2H-tetrazolium-5-carboxyanilide) sodium salt (Sigma-Aldrich) plus coenzyme Q_0_ (2,3-dimethoxy-5methyl-1,4-benzoquinone; Sigma-Aldrich) as previously described [6]. In brief, NK cells at an E:T ratio of 20:1 (based on the number of *Aspergillus* conidia used for the formation of hyphae) and various concentrations of immunosuppressive compounds were added to *A. fumigatus* hyphae for 6 hours. NK cells alone served as control. Thereafter, NK cells were lysed with sterile aqua dest., and hyphae were incubated in an XTT solution (0.25 mg/mL) supplemented with coenzyme Q_0_ (40 µg/mL) at 37 °C for 1 hour. The absorbance of the supernatant was then assessed spectrophotometrically at 450 nm using a 690-nm reference. Hyphal damage was calculated as follows: Percent hyphal damage = (1-X/C) × 100, where X is the absorbance of experimental wells and C is the absorbance of control wells with hyphae only. Each experiment has been performed at least three times, and all experiments have been performed independently from each other using NK cells from a different donor.

### 4.5. Assessment of the NK Cell Proliferative Capacity

The effect of immunosuppressive agents on the proliferative capacity of human NK cells was assessed by means of the carboxy-fluorescein-diacetate-succinimidyl-ester labelling assay (CFSE; Molecular Probes, Eugene, OR, USA). NK cells cultivated in the absence of immunosuppressive drugs served as control. The percentage of CFSE-positive NK cells and CFSE mean fluorescence intensity (MFI), both of which decrease in proliferating cells, were assessed in CD56^+^CD3^-^ NK cell populations on days 0, 1, 3 and 7 by flow cytometry (Canto II, Beckton Dickinson; BD FACSDiva Software v6.1.3, BD Biosciences) [27]. The estimation of the number of NK cells was performed on day 7 in a Neubauer slide (LO-Laboroptik) using trypan blue staining. Each experiment has been performed at least three times, and all experiments have been performed independently from each other using NK cells from a different donor.

### 4.6. Assessment of NK Cell Viability

The effect of immunosuppressive compounds on apoptosis and necrosis of human NK cells was assessed by means of Annexin V and propidium iodide (PI) staining (BD Biosciences). NK cells were incubated with or without the immunosuppressive agent for 24 h and subsequently labeled according to manufacturer´s instructions. The percentages of annexin^+^PI^-^ (apoptotic) and annexin^+^PI^+^ (necrotic) NK cells, respectively, were assessed by flow cytometry (Canto II, Beckton Dickinson; BD FACSDiva Software v6.1.3, BD Biosciences). Each experiment has been performed at least three times, and all experiments have been performed independently from each other using NK cells from a different donor.

### 4.7. Assessment of the Concentration of Soluble Molecules in the Supernatant

Supernatants were collected after the NK cells had been incubated for six hours with or without *A. fumigatus* and with or without immunosuppressive compounds, respectively. Levels of RANTES (level of detection 0.002 pg/mL), granulocyte-macrophage colony-stimulating factor (GM-CSF) (0.2 pg/mL), and interferon (IFN)-γ (0.8 pg/mL) were analyzed by means of the Cytometric Bead Array (CBA; BD Biosciences) according to the manufacturer’s instructions. Levels of perforin (limit of detection 31.6 pg/mL) were assessed by means of ELISA (MABTECH AB, Nacka Strand, Sweden) according to the manufacturer’s instructions. Each experiment has been performed at least three times, and all experiments have been performed independently from each other using NK cells from a different donor.

### 4.8. Statistical Analyses

Data were analyzed using GraphPad Prism (version 5.04; GraphPad Software, La Jolla, CA, USA). Student´s t-test was used to compare each concentration of the respective immunosuppressant with the control (NK cells and *A. fumigatus*). A two-sided P value of less than 0.05 was considered to be statistically significant.

## Supplementary Materials

The following are available online at https://www.mdpi.com/2076-0817/8/4/246/s1, Figure S1: Measured anti-*Aspergillus* activity of immunosuppressive compounds.
