# Supplementary figures and images for: Distinct Effects of Immunosuppressive Drugs on the Anti-Aspergillus Activity of Human Natural Killer Cells
Source: Pathogens. 2019 Nov 19;8(4):246. doi: 10.3390/pathogens8040246 (PMC6963337; doi:10.3390/pathogens8040246)

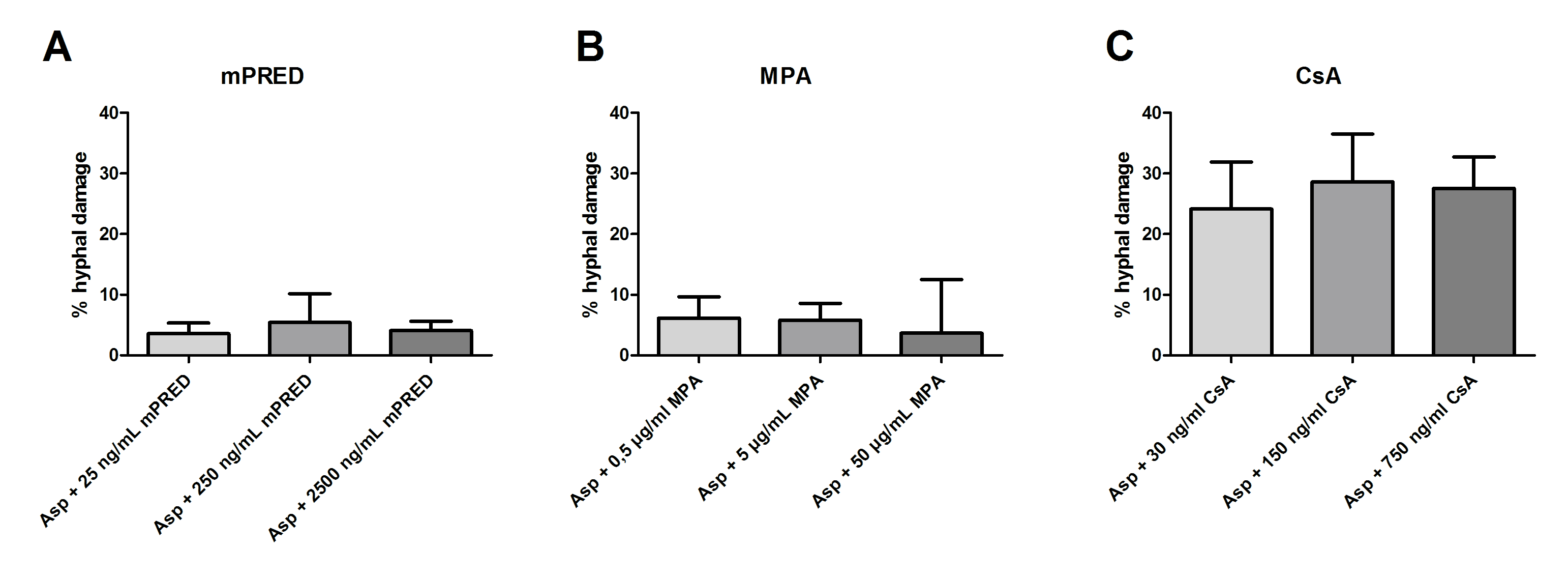

Supplement: Supplementary file 1 [file pathogens-08-00246-s001.zip › pathogens-623270-supplementary.tif]
